# Supplementary material for: P-TRAP: a Panicle Trait Phenotyping tool
Source: BMC Plant Biol. 2013 Aug 29;13:122. doi: 10.1186/1471-2229-13-122 (PMC3848748; doi:10.1186/1471-2229-13-122)
Supplement: Additional file 7 — P-TRAP source code. The Netbeans project that contains the source code of the application. [file 1471-2229-13-122-S7.zip › souce.zip!/P-TRAP source code/Grain/doc/javadoc/org-bioalg-configurationfilesprocessor/org/bioalg/configurationfilesprocessor/XMLConfigurationParser.html]

XMLConfigurationParser (ConfigurationFilesProcessor)


JavaScript is disabled on your browser.


- Package
- Class
- Tree
- Deprecated
- Index
- Help

*org.bioalg.configurationfilesprocessor 1.0*

- Prev Class
- Next Class

- Frames
- No Frames

- All Classes

- Summary:
- Nested |
- Field |
- Constr |
- Method

- Detail:
- Field |
- Constr |
- Method


org.bioalg.configurationfilesprocessor

## Class XMLConfigurationParser

- java.lang.Object
- - org.bioalg.configurationfilesprocessor.XMLConfigurationParser

- ---

    

  ```
  public abstract class XMLConfigurationParser
  extends java.lang.Object
  ```

  Author:
  :   faroq

- - ### Constructor Summary

    Constructors

    Constructor and Description

    `XMLConfigurationParser()`
  - ### Method Summary

    Methods

    | Modifier and Type | Method and Description |
    | --- | --- |
    | `static int` | `getIntValue(org.w3c.dom.Element ele, java.lang.String tagName)` |
    | `static java.lang.String` | `getTextValue(org.w3c.dom.Element ele, java.lang.String tagName)` |
    | `static org.w3c.dom.Document` | `getXMLDocument(java.io.File file)` |
    | `static org.w3c.dom.Document` | `getXMLDocument(java.io.InputStream string)` |

    - ### Methods inherited from class java.lang.Object

      `clone, equals, finalize, getClass, hashCode, notify, notifyAll, toString, wait, wait, wait`

- - ### Constructor Detail


    - #### XMLConfigurationParser

      ```
      public XMLConfigurationParser()
      ```
  - ### Method Detail


    - #### getXMLDocument

      ```
      public static org.w3c.dom.Document getXMLDocument(java.io.File file)
      ```


    - #### getXMLDocument

      ```
      public static org.w3c.dom.Document getXMLDocument(java.io.InputStream string)
      ```


    - #### getTextValue

      ```
      public static java.lang.String getTextValue(org.w3c.dom.Element ele,
                                  java.lang.String tagName)
      ```


    - #### getIntValue

      ```
      public static int getIntValue(org.w3c.dom.Element ele,
                    java.lang.String tagName)
      ```


- Package
- Class
- Tree
- Deprecated
- Index
- Help

*org.bioalg.configurationfilesprocessor 1.0*

- Prev Class
- Next Class

- Frames
- No Frames

- All Classes

- Summary:
- Nested |
- Field |
- Constr |
- Method

- Detail:
- Field |
- Constr |
- Method
